# Role of Alternative Medical Systems in Adult Chronic Kidney Disease Patients: A Systematic Review of Literature

**DOI:** 10.7759/cureus.32874

**Published:** 2022-12-23

**Authors:** Wei Yi Teo, Shu Wen Felicia Chu, Li Yue Chow, Cheng Teng Yeam, Lian Leng Low, Joanne Hui Min Quah, Marjorie Foo, Jun Jie Benjamin Seng

**Affiliations:** 1 Biological Sciences, National University of Singapore, Singapore, SGP; 2 Anesthesiology and Perioperative Medicine, Singapore General Hospital, Singapore, SGP; 3 Accounting, Singapore Management University, Singapore, SGP; 4 Anesthesiology, Ministry of Health Holdings, Singapore, SGP; 5 Family Medicine and Continuing Care, Singapore General Hospital, Singapore, SGP; 6 Family Medicine, SingHealth Polyclinics, Singapore, SGP; 7 Renal Medicine, Singapore General Hospital, Singapore, SGP; 8 Family Medicine, Singapore Health Services, Singapore, SGP; 9 Medicine, Ministry of Health Holdings, Singapore, SGP

**Keywords:** renal insufficiency, chronic kidney diseases, complementary therapies, therapeutics, alternative medical systems, systematic reviews

## Abstract

There is a growing interest in the use of alternative medical systems (AMS), such as traditional Chinese medicine (TCM), ayurveda, homeopathy, and naturopathy, among chronic kidney disease patients. This review summarizes the efficacy and safety of AMS interventions in chronic kidney disease (CKD) patients. A systematic review was conducted in MEDLINE, Embase, Scopus, CINAHL, CENTRAL, and PsycINFO in line with the Preferred Reporting Items for Systematic Reviews and Meta-Analyses (PRISMA) and Synthesis without meta-analysis (SWiM) guidelines. Randomized controlled trials (RCTs) which evaluated the use of AMS among adult CKD patients were included. The efficacy of each AMS was assessed based on improvement in biochemical markers or reduction in symptom severity scores. All adverse reactions were recorded. Of the 14,583 articles retrieved, 33 RCTs were included. TCM (n=20) and ayurveda (n=6) were the most well-studied. Majority of studies (66.7%) had a sample size <100. Common indications evaluated included improvement in renal function (n=12), proteinuria (n=5), and uremic pruritus (n=5). Among TCM, acupuncture and syndromes-based TCM granules formulation were shown to improve estimated glomerular filtration rate (eGFR) by 5.1-15.5% and 7.07-8.12% respectively. Acupuncture reduced uremic pruritus symptoms by 54.7-60.2% while Huangkui, Shenqi granules, and *Tripterygium wilfordii* Hook F reduced proteinuria by 18.6-50.7%, 61.8%, and 32.1% respectively. For Ayurveda, camel milk and *Nigella sativa* oil improved eGFR by 16.9% and 86.8%, respectively, while capsaicin reduced pruritus scores by 84.3%. Homeopathic verum medication reduced pruritus scores by 29.2-41.5%. Nausea was the most common adverse effect reported with alpha-keto amino acids (0.07%), *Nigella sativa* oil (7.04%), and silymarin (10%). TCM and ayurveda were more well-studied AMS therapies that demonstrated efficacy in CKD patients. RCTs with larger sample sizes are needed to ascertain the efficacy and safety of promising AMS.

## Introduction and background

Chronic kidney disease (CKD) is one of the leading causes of death globally, which affects 13.4% of the world’s population [1]. With deterioration in renal function, this leads to the onset of CKD-related complications, such as uremia, anemia, and electrolyte disorders [2]. These complications often manifest as symptoms ranging from pruritus, pain, and insomnia to muscle cramps. This in turn has negative implications on patients’ quality of life [3,4]. Importantly as CKD patients approach end-stage renal disease (ESRD), the prevalence and severity of such symptoms increase [5].

Despite medical breakthroughs and the advent of new therapies in the past decades, optimal treatments for some of the symptoms resulting from CKD-related complications remained unclear, possibly due to their complex pathophysiology. A case in point is uremic pruritus, which is found in around 20% of pre-dialysis CKD patients and 40% of ESRD patients [6]. Although prevalent treatments include the use of emollients, gabapentin, and antihistamines, data related to their efficacy were often derived from small studies and their use is limited by adverse effects [7].

The use of alternative medical systems (AMS) which forms a key pillar of complementary and alternative medicine (CAM) has increased in the past 20 years [8]. AMS is defined as “entire systems of health theory and practice that developed separately from conventional medicine” [9]. Notably, around 18% of dialysis patients have utilized some form of AMS [10,11]. In addition, prescription of AMS therapies such as traditional Chinese medicine (TCM) by professional practitioners often aids in minimizing the risk of side effects, hence increasing their appeal as potential therapeutic alternatives [11].

Prior studies have shown that AMS is effective in reducing symptoms such as pain, nausea, and fatigue in non-CKD patient populations. For instance the use of TCM formulas, such as Liu Junzi Tang and Xiao Banxia Plus Fuling have demonstrated efficacy in treating cancer-related pain and chemotherapy-related nausea and vomiting [12]. In addition, Chinese herbs such as *Curcuma longa* and *Panax ginseng* among patients with malignancies have shown efficacy in promoting apoptosis of cancer cells and inhibiting tumor metastasis [13]. Another study showed that a multi-modal Ayurvedic treatment approach was effective in reducing knee osteoarthritis symptoms, such as pain and stiffness, and improving function [14]. With increasing research supporting the use of AMS, this has led to a rise in healthcare institutions adopting and providing such integrated services which are supported by insurance coverage [15].

Among CKD patients, multiple studies have also been conducted to assess the efficacy of AMS in the treatment of CKD-related conditions and symptoms such as uremic pruritis and anemia. For instance, a study that assessed the efficacy of homeopathy verum among CKD patients showed a reduction in pruritus symptoms by 49% after 30 days of treatment [16]. Another study that evaluated the use of TCM patients with glomerulonephritis showed improvement in hemoglobin after 24 weeks of therapy [16,17].

Existing reviews which have assessed the role of AMS are currently limited to specific indications, such as uremic pruritus [18], use of subtypes of AMS in specific CKD subgroups, such as consumption of Chinese herbal medicine in diabetic kidney disease [19], and specific AMS therapies, such as use of Astragalus [20,21]. This review aimed to summarize and evaluate the broad roles and efficacy of AMS as potential alternative therapeutic options for CKD patients. Findings from the review will aid physicians in gaining a better understanding of the efficacy of AMS for CKD patients, which can aid in facilitating purposeful discussions with patients who are using or considering these therapies.

## Review

Methods

Protocol and Registration

The protocol for this study was registered on Open Science Framework (https://osf.io/ymks8/) and was composed based on the Preferred Reporting Items for Systematic Reviews and Meta-Analyses (PRISMA) statement and Synthesis without Meta-Analysis (SWiM) reporting guidelines [22,23].

Information Sources and Search

A literature search was conducted in MEDLINE, Embase, Scopus, CENTRAL, CINAHL, and PsycINFO. There was no start date restriction, and studies up to April 2022 were included. Key terms related to CKD, randomized controlled trials (RCT), and AMS were included in the searches. The search terms were adapted from other systematic reviews and the full search strategy is available in Appendix 1 [18,24-26].

Eligibility Criteria

With regards to inclusion criteria, full-text articles in English language which involved RCTs evaluating the use of AMS in adult CKD patients (>18 years old) were included. As defined by the National Center for Complementary and Integrative Health (NCCIH), AMS is a broad category encompassing a variety of medical modalities and refers to an entire system of theory and practice which developed separately from conventional medicine [15]. In this review, we included TCM, naturopathy, homeopathy, and ayurvedic medicine. Non-RCTs, case series, other systematic reviews, and meta-analyses were excluded.

Description of Main Types of AMS

TCM: TCM is a system of medical practice which originated in China and adopts a holistic approach to the medical treatment of a patient based on “syndrome differentiation.” It focuses on the integrity of the human body by emphasizing the intimate relationship between the body and its social and natural environment, as well as dynamic balance of movement [27]. The basic tenet of TCM is based on the flow and balance of vital energy, Qi, which flows through channels in the body called meridians that connect various organs and tissues [28]. Diseases are believed to be brought about by the imbalance of Qi. Hence, by restoring balance via acupoints or intake of herbs, TCM seeks to promote individual wellness and prevent diseases [29].

Naturopathy: Naturopathy is a form of medical practice which is rooted in vitalism and folk medicine and, promotes natural and self-healing ideologies [30]. The unique attribute of naturopathic medicine lies in the reprioritization of the order of therapeutics with increased emphasis on preventive behaviors, lifestyle modifications, nutrition, and exercise, over medical or surgical interventions [31].

Homeopathy: Homeopathy entails the therapeutic administration of substances derived from plants, minerals, or animals that produce effects that correspond to the clinical manifestation of diseases [32]. Its practice is centered on two following theories: “like cures like” and “law of minimum dose.” “Like cures like” refers to the belief that diseases can be treated with substances that produce similar symptoms in healthy individuals, and “law of minimum dose” refers to the belief that the lower the dose the greater its therapeutic efficacy [32].

Ayurvedic medicine: Ayurvedic medicine is one of the oldest alternative medical systems which involves the use of therapeutics derived predominantly from plants, animals, minerals, diet, exercise, and lifestyle changes. Its therapies are centered on the principle of “Panchakarma,” which comprises five karmas (actions) to rejuvenate and remove toxins from one’s body [33].

Study Selection and Data Collection Process

Citations retrieved from the six databases were extracted into Endnote X9 software (Philadelphia, PA: Clarivate) and duplicated citations were removed. During the initial article screening, two independent reviewers (WY and SW) reviewed the titles and abstracts of articles to select relevant articles. Thereafter, the full texts of the identified articles were evaluated. All discrepancies during the article screening process were resolved by discussion with a third reviewer (JJ). Hand-searching of references within identified articles was also performed to enhance the comprehensiveness of the search. A standardized Microsoft Excel data collection form was used for data extraction, and details related to the study characteristics, studied indications of intervention, efficacy, and safety were collected.

Management of Missing Data

For studies with missing data, authors were contacted for clarification to enhance the comprehensiveness of this review. Missing information that could not be retrieved after two email reminders were labeled as unavailable.

Risk of Bias in Individual Studies

The Cochrane Risk of Bias tool version 2.0 (Oxford, England: Cochrane) was utilized in the assessment of the included RCTs [34]. Two independent reviewers (Teo and Chu) performed the risk of bias assessment (Appendix 2). The instrument comprises of the following five domains: risk of bias arising from the randomization process, deviations from intended interventions, missing outcome data, outcome measurement, and selection of reported results. Using the responses derived from the five domains, the overall risk of bias for the individual studies was rated as "low," "some concerns," or "high" risk of bias.

Assessment of Heterogeneity

Clinical and methodological heterogeneity of included studies were analyzed to evaluate if meta-analyses could be performed for specific interventions in this study. Clinical heterogeneity describes variation in characteristics of study participants, intervention, or outcomes while methodological heterogeneity describes variation in study design and risk of bias. This was performed by two independent reviewers (Yeam and Seng). In view of the clinical and methodological heterogeneity across the included studies, a narrative review of the RCTs was conducted.

Synthesis of Data

With regard to the efficacy of AMS interventions, the response rates and any changes in patients’ quality of life were recorded. Additionally, the safety profile of each intervention was evaluated and the reported prevalence, severity, and outcomes of adverse effects were tabulated. The adverse events reported in included studies were categorized using the Common Terminology Criteria for Adverse Events (CTCAE) [35].

Summary Measures

Descriptive statistics were utilized to summarize the characteristics of all included studies. The principal summary measures evaluated in this study were the studied indications, efficacy of each AMS, and safety profile of each AMS.

Results

Study Selection

**Figure 1:**
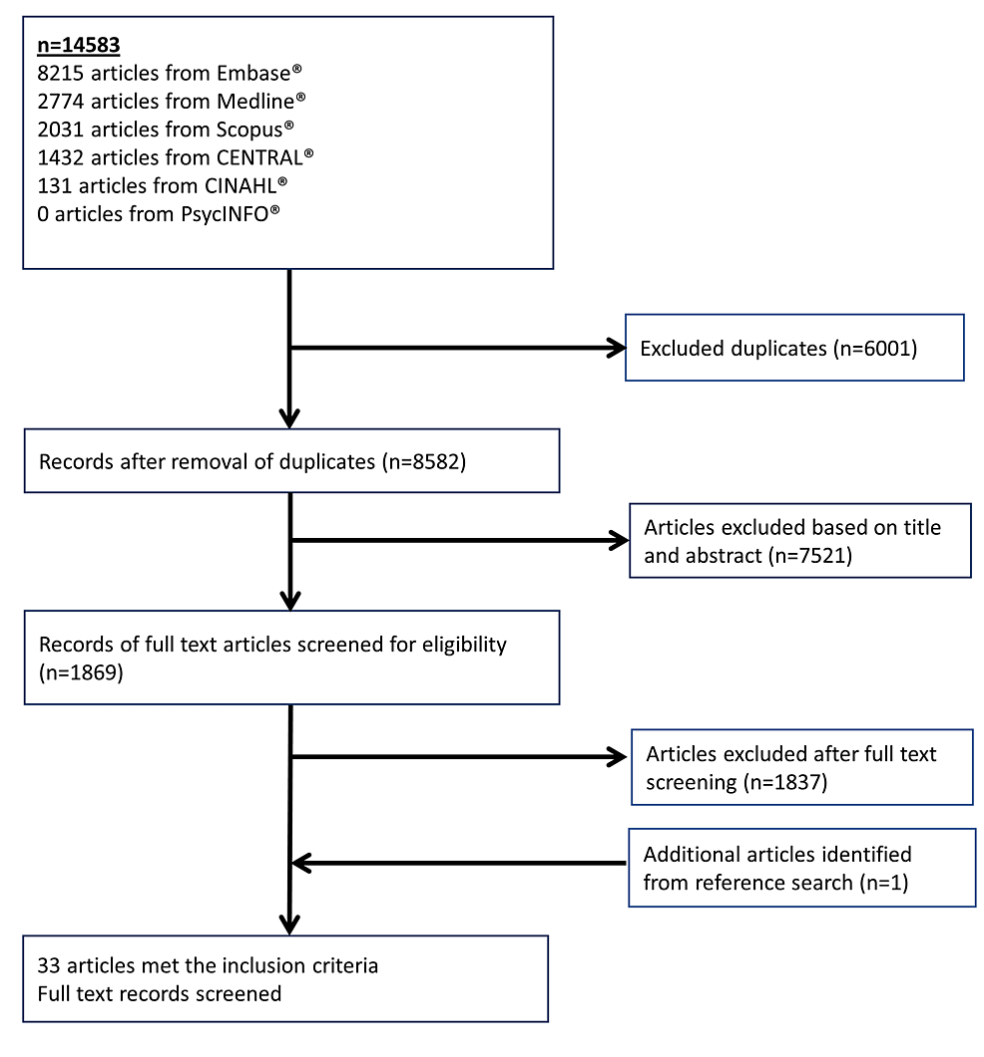
Flow chart for the inclusion of articles in this systematic review.

Out of 14,583 retrieved citations, 33 full-text articles were included in this review. The inclusion and exclusion criteria for the studies are shown in Figure 1. The percentage of agreement of articles between the reviewers was 94.0% and all disagreements were resolved after discussion.

Study Characteristics

Types of AMS studied: Table 1 shows the characteristics of included studies. Among the four main types of AMS, TCM was the most studied (n=20, 60.6%) [3,7,17,36-52], followed by Ayurveda (n=6, 18.2%) [53-58], naturopathy (n=5, 15.2%) [59-63], and homeopathy (n=2, 6.1%) [16,64].

**Table 1:** Characteristics of included AMS intervention studies. ^a^QDDHG is composed of Huang Qi, Danshen, Dihuang, Shanyao, and Gan Cao. ^b^Dosage and frequency vary from patient to patient. ARB: angiotensin receptor blockers; DN: diabetic neuropathy; ESRD: end-stage renal disease; TBN: tianbaoning; TSF: tangshen formula; TWHF: Tripterygium wilfordii Hook F; ZSTL: zishentongluo; QoL: quality of life; QD: once a day; TIW: thrice a week; TID: thrice a day; CKD: chronic kidney disease; QIW: four times a week; HD: hemodialysis; T: test group; C: control group; T1: test group 1; T2: test group 2; C1: control group 1; C2: control group 2

Study (year of publication)	Study design	Number of patients in treatment and control arm	Indication for use of intervention	Treatment	Comparator	Country	Patient population	Mean age of patients (SD)	Gender (male) (%)
Traditional Chinese medicine (n=20)									
Li et al. (2015) [41]	Double-blinded parallel arm	T_1_:66, C_1_:32, T_2_:56, C_2_:26	Renal function	8 g TSF granules and ARB BID x 24 weeks	8 g placebo TID and ARB BID x 24 weeks	China	Non-dialysis CKD patients	T_1_:59.5 (10.1), C_1_:56.7 (9.3), T_2_:58.9 (9.0), C_2_:60.8 (10.0)	T_1_:54.5%, C_1_:53.1%, T_2_:58.9%, C_2_:53.8%
Ma et al. (2013) [43]	Parallel arm	T:25, C:20	Renal function	150 mL ZSTL solution BID x 3 months	10 mg benazepril QD x 3 months	China	Early DN patients	T:57, C:57	T:40%, C:40%
Wang et al. (2012) [17]	Double-blinded parallel arm	T_1_:192, T_2_:191, C:189	Renal function	T1: TCM granules BID x 24 weeks, T_2_: TCM granules BID and 10mg benazepril QD x 24 weeks	10 mg benazepril QD and TCM placebo TID x 24 weeks	China	CKD stage 3	T_1_:47.3 (10.9), T_2_:49.3 (11.4), C:49.0 (10.5)	T_1_:54.7%, T_2_:47.1%, C:47.6%
Yu et al. (2017) [50]	Single-blinded parallel arm	T:28, C:25	Renal function	Acupuncture at Li4, ST36 and K13 acupoint QD x 3 months	Sham acupuncture QD x 3 months	China	CKD stage 2-4	T:58.5, C:61.0	T:89.3%, C:88.0%
Zhao et al. (2020) [52]	Double-blinded parallel arm	T:171, C:172	Renal function	Herbal granules TID x 6 months	Placebo granules x TID 6 months	China	CKD stage 3	T:51.89 (13.12), C:52.03 (12.62)	T:62.0%, C:70.4%
Xiang et al. (2016) [47]	Parallel arm	T:51, C:51	Renal function	QDDHG^a^ tablets BID x 12 weeks	ARB tablets (minimum dosage)	China	DN patients	T:57.21 (13.20), C:58.16 (11.59)	T:24%, C:22%
Xu et al. (2016) [49]	Double-blinded parallel arm	T:91, C:86	Renal function	500 mg GS-Rb1 (ginseng extract) QD x 6 months	Placebo tablets QD x 6 months	China	CKD stage 2-3	T:59.2 (8.5), C:58.4 (7.5)	T:72.5%, C:70.9%
Chen et al. (2013) [37]	Parallel arm	T:95, C:95	Proteinuria	9.6 g of Shenqi particle TID x 48 weeks	Routine care	China	Non-dialysis CKD patients	T:49 (14), C:53 (12)	T:36.2%, C:68.42%
Ge et al. (2013) [39]	Parallel arm	T:34, C:31	Proteinuria	40 mg TWHF TID x 3 months followed by 20 mg TWHF TID x 3 months	80 mg valsartan BID x 6 months	China	DN patients	T:51.9 (9.8), C:51.0 (8.9)	T:58.8%, C:54.8%
Li et al. (2020) [42]	Double-blinded parallel arm	T:735, C:735	Proteinuria	Huangkui capsule TID x 12 months	Losartan potassium tablet QD and placebo capsules TIW x 12 months	China	CKD stage 1-3a	T:37.7 (10.9), C:37.1 (10.4)	T:48.0%, C:46.3%
Zhang et al. (2014) [51]	Parallel arm	T_1_:133, T_2_:136, C:135	Proteinuria	T_1_: Huangkui capsule TID x 6 months, T_2_: Huangkui capsule TID and Losartan potassium tablet QD x 6 months	Losartan tablet potassium QD x 6 months	China	Non-dialysis CKD patients	T_1_:37.3 (12.5), T_2_:37.1 (11.1), C:38.1 (12.7)	T_1_:50.4%, T_2_:47.1%, C:53.3%
Xiong et al. (2020) [48]	Parallel arm	T:62, C:62	Proteinuria	60 mg TWHF and 160 mg valsartan QD x 24 weeks	160 valsartan QD x 24 weeks	China	Non-dialysis CKD patients	T:50.3 (11.8), C:49.6 (12.3)	T:69.4%, C:72.6%
Che-Yi et al. (2005) [36]	Double-blinded parallel arm	T:20, C:20	Uremic pruritus	Acupuncture at Quchi (L11) acupoint TIW x 1 month	Sham acupuncture TIW x 1 month	China	ESRD	T:62.4 (9.1), C:63.2 (7.5)	T:45.0%, C:50.0%
Gao et al. (2002) [38]	Double-blinded parallel arm	T:34, C:34	Uremic pruritus	Acupuncture at Quchi (L11) and Zusanli (ST 36) acupoint BIW x 1 month	Sham acupuncture BIW x 1 month	China	ESRD	43.6	59.0%
Nahidi et al. (2018) [7]	Single-blinded parallel arm	T:15, C:11	Uremic pruritus	Acupuncture at various acupoints TIW x 6 weeks	Sham acupuncture TIW x 6 weeks	Iran	HD	T:54.7 (11.4), C:41.4 (16.2)	T:60%, C:73%
Ono et al. (2015) [3]	Parallel arm	T:23, C:17	Fatigue, insomnia, itchiness, and pain	Acupuncture QIW x 2 months	Routine care	Japan	HD	T:70.0 (9.6), C:67.3 (13.0)	NI
Su et al. (2009) [44]	Parallel arm	T:31, C:30	QoL	Infrared stimulation of Qihai (RN6), Kuamyuan (RN4) and Chungchi (RN3) TIW x 3 months	Heat pad therapy to acupoints TIW x 3 months	China	ESRD	T:61.07 (13.9), C:58.6 (12.6)	T:51.6%, C:56.7%
Wang et al. (2019) [45]	Single-blinded parallel arm	T:40, C:40	Wnt/β-catenin signaling pathway	Qingshen granules TID x 3 months	Placebo granules TID x 3 months	China	CKD stage 3-5	T:52.1 (10.4), C:54.9 (9.2)	T:55%, C:48%
Wang et al. (2020) [46]	Parallel arm	T:136, C:146	Immune function	Qingshen granules TID x 3 months	Routine care	China	CKD stage 3-5	T:54.0 (10.5), C:51.8 (12.0)	T:55.1%, C:57.5%
Li et al. (2009) [40]	Parallel arm	T:32, C:32	Vascular endothelial function	TBN tablets (gingko extract) TI x 8 weeks	Routine care	China	Early DN patients	T:66.5 (71.1), C:67.2 (7.2)	T:53.1%, C:50%
Ayurveda (n=6)									
Alam et al. (2020) [53]	Parallel arm	T:70, C:66	Renal function	Sativa oil QD and alpha-keto amino acid tablets TID x 3 months	Alpha-keto amino acid tablets TID x 3 months	India	CKD stage 3-4	T:49.2, C:48.8	T:58.6%, C:51.5%
Fallahzadeh et al. (2012) [54]	Double-blinded parallel arm	T:30, C:30	Renal function	140 mg silymarin tablet QD x 3 months	Placebo tablet QD x 3 months	Iran	Non-dialysis CKD patients	T:55.9 (8.3), C:57.6 (7.5)	T:50%, C:43.3%
Hoseini et al. (2019) [55]	Parallel arm	T:22, C:22	Renal function	Camel milk BID x 3 months	Routine care	Iran	CKD stage 3-4	56.7 (11.8)	52.3%
Khajehdehi et al. (2011) [56]	Double-blinded parallel arm	T:28, C:28	Renal function	140 mg silymarin TID x 3 months	Placebo tablet TID x 3 months	Iran	Non-dialysis CKD patients	T:55.9 (8.3), C:57.6 (7.5)	T:50%, C:43.3%
Makhlough et al. (2010) [57]	Double-blinded parallel arm	T:17, C:17	Uremic pruritus	0.03% capsaicin ointment QID x 4 weeks	Placebo ointment QID x 4 weeks	Iran	ESRD	57 (18.6)	41.2%
Pingali et al. (2020) [58]	Double-blinded parallel arm	T_1_:18, T_2_:18, C:19	Hyperuricemia	T_1_:500 mg of beleric capsule taken QD T_2_: 1000 mg of beleric capsule taken QD	C:40 mg of febuxostat taken QD	India	CKD stage 2-3	T_1_:53.2 (8.9), T_2_:50.8 (8.8), C:51.0 (9.8)	T_1_:72.2%, T_2_:77.8%, C:73.7%
Naturopathy (n=5)									
Khan et al. (2014) [60]	Double-blinded parallel arm	T:80, C:80	Malnutrition	Alpha-keto amino acid tablets TID x 3 months	Placebo tablets TID x 3 months	India	Non-dialysis CKD patients	T:45.0, C:45.0	T:59.5%, C:57.8%
Prakash et al. (2004) [61]	Double-blinded parallel arm	T:21, C:19	Malnutrition	Keto amino acid tablets QD x 9 months	Placebo tablets QD x 9 months	India	CKD stage 3-4	T:52.8 (14.1), C:55.9 (17.6)	T:55.6%, C:43.8%
Sedaghattalab et al. (2021) [62]	Double-blinded parallel arm	T:22, C:23	Inflammation	Watercress extract QD x 1 month	Placebo extract QD x 1 month	Iran	HD	T:58.9 (16), C:63.1 (13)	NI
Zare et al. (2019) [63]	Double-blinded parallel arm	T:19, C:21	Inflammation	Garlic extract tablets TIW x 2 months	Placebo tablets TIW x 2 months	Iran	PD	T:56.0 (16.1), C:52.8 (18.8)	T:42.1%, C:42.8%
Boldaji et al. (2019) [59]	Crossover trial	T:22, C:19	Hypertension, stress, and inflammation	Pomegranate juice TIW x 2 months	Routine care	Iran	ESRD	47.8 (13.3)	61%
Homeopathy (n=2)									
Cavalcanti et al. (2003) [16]	Double-blinded parallel arm	T:11, C:9	Uremic pruritus	Homeopathic verum medication^b^ administered	Placebo medication administered	Brazil	HD	T:47, C:57	T:64%, C:56%
Silveira et al. (2019) [64]	Double-blinded parallel arm	T:18, C:14	Renal function	Brazilian green propolis pills BID x 3 months	Placebo pills BID x 3 months	Brazil	CKD stage 1-5	T:52.8 (14.1), C:55.9 (17.6)	T:55.6%, C:43.8%

Overview of study design and patient characteristics in included studies are as follows: the majority of the studies were conducted in Asia (n=31, 93.9%), whereas most of the studies were performed in China (n=18, 54.5%). Out of all reviewed studies, 20 (60.6%) were blinded to randomized controlled trials. The sample size was greater than 50 patients in 21 (63.6%) trials (Table 1). Non-dialysis patients were recruited in seven studies (21.2%), whereas ESRD patients were recruited in five studies (15.2%). The average duration of follow-up for all studies was 4.9 months (Table 2).

**Table 2:** Assessment of outcomes and efficacy of different AMS interventions. ^a^Percentage reduction in symptoms is computed using (mean score at baseline - mean score post-treatment at end of study)/(mean score at baseline) for the treatment group. ^b^TCM granules are formulated based on four TCM syndrome patterns (Qi Yin/Xue deficiency, blood stasis in the kidney, wind-dampness interfering in the kidney, and endoretention of damp heat) for the treatment of CKD. ^c^Herbal granules consist of ten herbs: Huangqi, Danggui, Huzhang, Liuyuexue, Tufuling, Niuxi, Shiwei, Dahuang, Jixuecao, and Huangjing. ^d^Dosage and frequency vary from patient to patient. 99mTc-DTPA: 99 m technetium diethylenetri-aminepenta-aceticacid; ARB: angiotensin II receptor blockers; BAID: brachial arterial inner diameter; BIW: twice a week; BUN: blood urea nitrogen; CCR: creatinine clearance rate; CKD-EPI: chronic kidney disease-epidemiology collaboration; DQOL: diabetes quality of life; eGFR: estimated glomerular filtration rate; ESR: erythrocyte sedimentation rate; ELISA: enzyme-linked immunosorbent assay; EQ-5D: EuroQol-5 dimension; FBG: fasting blood glucose; GS-RB1: Ginsenoside Rb1; HbA1c: haemoglobin A1c; ITT: intention to treat; LF: low frequency; MDA: malondialdehyde; MDRD: modification of diet in renal disease; NI: no information; PP: per protocol; QD: once a day; QDDHG: Qidan Dihuang Grain; SCr: serum creatinine; TBN: Tianbaoning; TCM: traditional Chinese medicine; TIW: thrice a week; TSF: Tangshen formula; TwHF: Tripterygium Wilfordii Hook F; TUP: total urine protein; TUV: total urine volume; UAER: urinary albumin excretion rate; VAS: visual analog scale; WHOQOL-BREF: World Health Organization Quality of Life-100 Questionnaire; ZSTL: Zishentongluo; Hs-CRP: high-sensitivity c-reactive protein; ITT: intention to treat; QIW: four times a week; HIF-1α: hypoxia-inducible factor 1-alpha; Wnt1: Wnt family member 1; α-SMA: alpha smooth muscle actin; TNF-α: tumor necrosis factor alpha; TRAF6: tumor necrosis factor receptor associated factor 6; FN: Fibronectin

Study (year of publication)	Indication for use of intervention	Treatment (dose and duration if available)	Comparator (dose and duration if available)	Tool(s) used to assess outcomes	Outcome	Improvement symptoms (yes / no)	Percentage reduction^a^/improvement in symptoms (if available)	Follow-up duration
Traditional Chinese medicine (n=20)								
Li et al. (2015) [41]	Renal function	8 g TSF granules TID and ARB BID x 24 weeks	8 g placebo TID and ARB BID x 24 weeks	WHOQOL-BREF, DQOL	UAER (μg/min) (pre vs post): 105.39±77.29 vs 88.37±108.46, p=0.021	Yes	-16.1% (UAER)	6 months
					24 h urinary protein (g/24 h) (pre vs post): 1.12±0.75 vs 0.91±0.90, p=0.017		-18.8% (24 h urinary protein)	
Ma et al. (2013) [43]	Renal function	150 mL ZSTL solution BID x 3 months	10 mg benazepril QD x 3 months	Radioimmunoassay, ELISA	HbA1c (%) (baseline vs mean change from baseline): 10.68 (8.48, 13.96) vs -4.29 (-5.85, -2.79), p<0.05.	Yes	-40.2% (HbA1c)	9 months
					UAER (μg/min) (baseline vs mean change from baseline): 211.52 (164.58, 243.89) vs -106.99 (-121.29, -85.55), p<0.05		-50.6% (UAER)	
					SCr (μmol/L) (baseline vs mean change from baseline): 87.17 (70.59, 110.25) vs -3.33 (-11.02, 2.15), p<0.05		-3.82% (SCr)	
					CCR (mL/min) (baseline vs mean change from baseline): 139.86 (129.58, 149.52) vs -9.22 (-13.42, -5.82), p <0.05		-6.59% (CCR)	
Wang et al. (2012) [17]	Renal function	T_1_: TCM granule^b^ BID x 24 weeks. T_2_: TCM granule^b^ BID and 10 mg benazepril QD x 24 weeks.	10 mg benazepril QD and TCM placebo TID x 24 weeks	MDRD study equation, TCM assessing sheets	eGFR (mL/min/1.73 m^2^) (pre vs post): T_1_: 45.26±10.12 vs 48.46±15.90, p<0.05. T_2_: 44.68±9.82 vs 48.31±17.50, p<0.05.	Yes	7.07% (eGFR; T_1_), 8.12% (eGFR; T_2_)	6 months
					24 h proteinuria (mg/24 h) (pre vs post): T_1_: 725.98 vs 990.00, p<0.05. T_2_: 590.00 vs 453.50, p<0.05		36.4% (proteinuria; T_1_), -21.1% (proteinuria; T_2_)	
					Urinary albumin/creatinine (mg/gCr) (pre vs post): T_2_: 0.30 vs 0.22, p<0.05		-26.7% (urinary albumin/creatinine; T_2_)	
					Hb (g/L) (pre vs post): T_1_: 127.31±18.47 vs 129.57±21.82, p<0.05		17.8% (Hb; T_1_)	
Yu et al. (2017) [50]	Renal function	Acupuncture at Li4, ST36 and K13 acupoint QD x 3 months	Sham acupuncture QD x 3 months	NI	SCr levels (mg/dL) (T vs C): baseline: 1.45 vs 1.67, p=0.1298. Post-intervention: 1.41 vs 1.65, p=0.0489. 3-month follow-up: 1.32 vs 1.81, p=0.0467	Yes	-2.76% (SCr; pre vs post), -9.00% (SCr; pre- vs 3 months follow-up)	6 months
					eGFR (mL/min/1.73m^2^) (T vs C): Baseline: 51.85 vs 42.50, p=0.0855. Post-intervention: 54.50 vs 43.60, p=0.0470. 3-month follow-up: 59.90 vs 40.80, p=0.0191		5.11% (eGFR; pre vs post), 15.5% (eGFR; pre-intervention vs 3 months follow-up)	
					hs-CRP (mg/dL) (T vs C): Baseline: 1.10 vs 0.79, p=0.4361. Post-intervention: 0.80 vs 0.90, p=0.8773		-27.3% (hs-CRP pre vs post)	
Zhao et al. (2020) [52]	Renal function	Herbal granule^c^ TID x 6 months	Placebo granules x TID 6 months	Dye-binding method, Cerebrospinal fluid protein test kit, Determiner L CRE kit	SCr (μmol/L) (pre vs weeks 16, 20 and 24): 148.42±35.90 vs 130.19±29.79, 130.08±30.57, 130.78±32.55, p<0.05	Yes	-12.3% (SCr; pre vs 16 weeks), -12.4% (SCr; pre vs 20 weeks), -11.9% (SCr; pre vs 24 weeks)	6 months
Xiang et al. (2016) [47]	Renal function	QDDHG tablets BID and ARB (minimum dosage) x 12 weeks	ARB tablets (minimum dosage)	Guidelines for clinical research of Chinese medicine	Albumin (mg/24h) (within treatment group, baseline vs 4 vs 8 vs 12 week): 85.30 (66.00, 176.30) vs 61.50 (49.00, 110.20), p<0.05 vs 51.00 (37.00, 90.00), p<0.05 vs 41.40 (29.00, 68.00), p<0.05	Yes	-27.9% (Albumin; 4 weeks), -40.2% (Albumin; 8 weeks), -43.9% (Albumin; 12 weeks)	3 months
					Proteinuria (g/24h) (within treatment group, baseline vs 4 vs 8 vs 12 week): 0.20 (0.10, 0.30) vs 0.10 (0.10, 0.20), p<0.05 vs 0.10 (0.10, 0.20), p<0.05 vs 0.10 (0.10, 0.20), p<0.05		-50% (Proteinuria; 4, 8, 12 weeks)	
					Albumin/creatinine (mg/mol) (within treatment group, baseline vs 4 vs 8 vs 12 week): 20.70 (11.00, 30.50) vs 16.30 (8.10, 25.00), p<0.05 vs 15.00 (7.20, 20.60), p<0.05 vs 10.10 (5.60, 17.00), p<0.05		-21.3% (albumin/creatinine; 4 weeks), -27.5% (albumin/creatinine; 8 weeks), -51.2% (albumin/creatinine; 12 weeks)	
Xu et al. (2016) [49]	Renal function	500 mg GS-Rb1 (ginseng extract) QD x 6 months	Placebo tablets QD x 6 months	ELISA	Creatinine and urea level (T vs C): 6 months, p<0.01. 12 months, p<0.01	Yes	-	12 months
					Oxidative stress markers (T vs C): 6 months, p<0.01. 12 months, p<0.05			
					TNF-a level (T vs C): 6 months, p<0.05			
Chen et al. (2013) [37]	Proteinuria	9.6 g of Shenqi particle TID x 48 weeks	Routine care	MDRD study equation	Proteinuria (g/d) (pre vs post): 5.34±2.74 vs 2.04±2.15, p<0.001	Yes	-61.8% (proteinuria)	12 months
					eGFR (mL/min/1.73 m^2^) (pre vs post): 84.6±27.0 vs 100.7±37.5, p=0.001		19.0% (eGFR)	
Ge et al. (2013) [39]	Proteinuria	40 mg TwHF TID x 3 months, 20 mg TwHF TID x 3 months.	160 mg valsartan capsules QD x 6 months	Trichloroacetic acid method, Jaffe reaction, MDRD study equation, high-performance liquid chromatography	Urinary protein (g/24 h) (pre vs 1 month, pre vs 3 months, pre vs 6 months): 4.99±2.25 vs 3.23±2.57, p<0.01. 4.99±2.25 vs 2.83±1.57, p<0.01. 4.99±2.25 vs 2.99±1.81, p<0.01	Yes	-35.3% (urinary protein; 1 months), -43.3% (urinary protein; 3 months), -40.1% (urinary protein; 6 months)	6 months
					eGFR (mL/min/1.73 m^2^) (pre vs 6 months): 43.07±21.65 vs 38.71±23.66, p<0.05		-10.1% (eGFR; 6 months)	
Li et al. (2020) [42]	Proteinuria	Huangkui capsule TID x 12 months	Losartan potassium tablet QD and placebo capsules TIW x 12 months	NI	Proteinuria (mg/24 h) (pre vs post): 1238.9±667.4 vs 1008.8±1104.7, p<0.001	Yes	-18.6% (proteinuria)	12 months
Zhang et al. (2014) [51]	Proteinuria	T_1_: Huangkui capsule TID x 6 months. T_2_: Huangkui capsule TID and Losartan potassium tablet QD x 6 months.	Losartan tablet potassium QD x 6 months	Biuret method, sarcosine oxidase assay	Proteinuria within T_1 _(pre vs 12 vs 24 weeks): 1045±420 vs 762±533, p<0.001 vs 537±409, p<0.001	Yes	T_1_: -27.1% (pre vs 12 weeks), -48.6% (pre vs 24 weeks)	6 months
					Proteinuria within T_2_ (pre vs 12 vs 24 weeks):1073±439 vs 783±658, p<0.001 vs 529±509, p<0.001.		T_2_: -27.0% (pre vs 12 weeks), -50.7% (pre vs 24 weeks).	
Xiong et al. (2020) [48]	Proteinuria	60 mg TWHF and 160 mg valsartan QD x 24 weeks	160 valsartan QD x 24 weeks	CKD-EPI equation	Proteinuria (g/24 h) (T vs C, PP analysis): 3.16±0.62 vs 4.28±0.85, p<0.001	Yes	PP: -26.2% (proteinuria)	6 months
					Serum albumin (g/L) (T vs C, PP analysis): 37.65±4.31 vs 33.59±4.56, p<0.001		PP: 12.1% (serum albumin)	
					Proteinuria (g/24 h) (T vs C, ITT analysis): 3.36±0.83 vs 4.52±1.06; p<0.001		ITT: -25.7% (proteinuria)	
					Serum albumin (g/L) (T vs C, ITT analysis): 36.91±4.42 vs 34.67±4.75, p=0.008		ITT: 6.46% (serum albumin)	
Che-yi et al. (2005) [36]	Uremic pruritus	Acupuncture at Quchi (L11) acupoint TIW x 1 month	Sham acupuncture TIW x 1 month	Validated questionnaire	Pruritus scores (pre vs post vs 3 months follow-up): 38.2±4.8 vs 17.3±5.5 vs 16.5±4.9, p<0.001	Yes	-54.7% (pruritus scores; pre- vs post-intervention), -56.8% (pruritus scores; pre-intervention vs 3 months follow-up)	3 months
Gao et al. (2002) [38]	Uremic pruritus	Acupuncture at Quchi (L11) and Zusanli (ST 36) acupoint BIW x 1 month	Sham acupuncture BIW x 1 month	NI	Number of patients (complete alleviation vs improvement vs no effect): 24 (70.6%) vs 9 (26.5%) vs 1 (2.9%)	Yes	-	3 months
Nahidi et al. (2018) [7]	Uremic pruritus	30 minutes of acupuncture, for six weeks, at the following acupoints: Sp6, Sp10, Lv3, Li4, Li11.	30 minutes of sham acupuncture, for 6 weeks.	VAS	Pruritus scores (pre vs post): 9.87±0.35 vs 3.93±2.85, p<0.001	Yes	-60.2% (pruritus scores)	6 weeks
Ono et al. (2015) [3]	Fatigue, insomnia, itchiness, and pain	Acupuncture QIW x 2 months	Routine care	VAS, EQ-5D	Headache score (pre vs post): 17.1±26.1 vs 6.2±13.5, p<0.05.	Yes	-63.7% (headache score)	3 months
					Blurred vision score (pre vs post): 33.4±32.7 vs 17.0±22.2, p<0.05.		-49.1% (blurred vision score)	
					Dizziness score (pre vs post): 13.0±21.4 vs 1.4±6.3, p<0.05.		-89.2% (dizziness score)	
					Ear buzzing (pre vs post): 17.9±27.2 vs 8.0±14.7, p<0.05		-55.3% (ear buzzing)	
					Cervical pain (pre vs post): 37.7±39.1 vs 25.3±29.7, p<0.05		-32.9% (cervical pain)	
					Stiff shoulders (pre vs post): 29.9±28.6 vs 12.5±21.6, p<0.05		-58.2% (stiff shoulders)	
					Back pain (pre vs post): 38.5±33.7 vs 9.3±18.1, p<0.05		-58.2% (back pain)	
					Lower limb pain (pre vs post): 29.4±36.4 vs 17.1±23.3, p<0.05		-41.8% (lower limb pain)	
					Numbness in upper limb (pre vs post): 18.9±30.4 vs 4.0±29.5, p<0.05		-78.8% (numbness in upper limb)	
					Numbness in lower limb (pre vs post): 21.9±34.9 vs 11.0±26.2, p<0.05		-49.8% (numbness in lower limb)	
					Itchiness (pre vs post): 38.7±40.7 vs 29.3±31.5, p<0.05		-24.3% (itchiness)	
					Difficulty in sleeping (pre vs post): 34.8±36.9 vs 12.8±22.5, p<0.05		-63.2% (difficulty in sleeping)	
					Utility in treatment group (pre vs post): 0.66±0.15 vs 0.76±0.17, p<0.05		15.2% (utility)	
Su et al. (2009) [44]	QoL	Infrared stimulation of Qihai (RN6), Kuamyuan (RN4) and Chungchi (RN3) TIW x 3 months	Heat pad therapy to acupoints TIW x 3 months	Heart rate variability analyser, WHOQOL-BREF questionnaire	LF activity (pre vs post): 49.99±79.08 vs 131.71±214.36, p=0.01	Yes	163% (LF activity)	3 months
					Fatigue index (pre vs post): 133.90±20.43 vs 121.71±32.68, p=0.02		-9.10% (fatigue index)	
					Psychological domain (pre vs post): 18.16±4.30 vs 19.39±0.72, p=0.02		6.77% (psychological domain)	
					Environmental domain (pre vs post): 29.87±4.04 vs 32.00±4.85, p=0.00.		7.13% (environmental)	
Wang et al. (2019) [45]	Wnt/β-catenin signaling pathway	Qingshen granules TID x 3 months	Placebo granules TID x 3 months	ELISA	Effective rates of TCM symptom (T vs C): 80% vs 60%, p=0.024	Yes	-	3 months
					eGFR (mL/min) (T vs C): 15.9±3.2 vs 14.0±4.0, p=0.019		17.8% (eGFR)	
					HIF-1𝛼 (ng/mL) (T vs C): 0.66±0.16 vs 1.39±0.17, p≤0.001		-61.4% (HIF-1𝛼)	
					Wnt1 (pg/mL) (T vs C): 314.2±85.8 vs 382.8±85.3, p=0.001		-16.9% (Wnt1)	
					𝛽-catenin (pg/mL) (T vs C): 416.5±13.6 vs 462.1±15.1, p ≤0.001		-10.0% (𝛽-catenin)	
					𝛼-SMA (KU/L) (T vs C): 20.5±3.1 vs 23.5±4.1, p≤0.001		-20.8% (𝛼-SMA)	
					E-cadherin (ng/mL) (T vs C): 2166.9±398.6 vs 2370.7±468.0, p=0.039		-15.1% (E-cadherin)	
Wang et al. (2020) [46]	Immune function	Qingshen granules TID x 3 months	Routine care	Flow cytometry, ELISA	CD4+/CD8+ T cell (pre vs post): 1.98±0.86 vs 1.58±0.72, p<0.05.	Yes	-20.2% (CD4+/CD8+ T cell)	3 months
					Th17 cell (pre vs post): 2.51±1.05 vs 1.70±0.83, p<0.01.		-32.3% (Th17)	
					NF-κB p65 (pre vs post): 36.84±12.96 vs 24.86±1.97, p<0.05		-32.5% (NF-κB p65)	
					IL-17 (pre vs post): 28.62±13.53 vs 19.78±12.25, p<0.05		-30.9% (IL-17)	
					IL-6 (pre vs post): 77.13±20.54 vs 58.42±18.25, p<0.05		-24.3% (IL-6)	
					TNF-α (pre vs post): 110.34±23.76 vs 75.49±22.80, p<0.01		-31.6% (TNF-α)	
					TRAF6 (pre vs post): 4.94±1.82 vs 2.85±1.53, p<0.01		-42.3% (TRAF6)	
					FN (pre vs post): 93.42±20.36 vs 62.86±19.35, p<0.01		-32.7% (FN)	
					Col-IV (pre vs post): 36.85±14.58 vs 24.36±13.36, p<0.01		-33.9% (Col-IV)	
					Total effective rate (T vs C): 79.41% vs 67.12%, p<0.05.		-	
Li et al. (2009) [40]	Vascular endothelial function	TBN tablets (gingko extract) TID x 8 weeks	Routine care	Chemical colorimeter, Radioimmunoassay, ELISA, Siemens Sequoia 512 color Doppler ultrasonography	UAER (μg/min) (pre vs post): 153.30±63.28 vs 85.15±36.82, p<0.01	Yes	-44.5% (UAER)	3 months
					SCr (μmol/L) (pre vs post): 120.76±17.83 vs 105.67±18.13, p<0.01		-12.5% (SCr)	
					NO (μmol/L) (pre vs post): 50.16±24.64 vs 70.65±28.71, p<0.01		40.8% (NO)	
					vWF (%) (pre vs post): 182.05±64.13 vs 128.56±48.98, p<0.01		-29.4% (vWF)	
					BAID responsive change (%) (pre vs post): 4.91±2.31 vs 6.78±3.89, p<0.01		38.1% (BAID responsive change)	
Ayurveda (n=6)								
Alam et al. (2020) [53]	Renal function	Sativa oil QD and alpha-keto amino acid tablets TID x 3 months	Alpha-keto amino acid tablets TID x 3 months	Hemogram, renal function test, serum electrolyte test	Hb% (g/dL) (pre vs post): 8.84±1.31 vs 10.24±1.10, p<0.001	Yes	15.8% (Hb%)	3 months
					24-h TUV (mL/day) (pre vs post): 1250.69±303.74 vs 1660.14±258.78, p<0.001		32.7% (TUV)	
					eGFR (mL/min) (pre vs post): 22.71±7.28 vs 42.42±17.38, p<0.001		86.8% (eGFR)	
Fallahzadeh et al. (2012) [54]	Renal function	140 mg silymarin tablet QD x 3 months	Placebo tablet QD x 3 months	Jaffé method, ELISA MDA assay, MDRD study equation, nephelometry, high-performance liquid chromatography, mercury sphygmomanometer	Urinary TNF-α (pg/mg) (change from baseline): -3.45 (-5.44 to -1.46), p<0.05	Yes	-	2 months
					Urinary MDA (nmol/mg) (change from baseline): -1.5 (-2.87 to -0.13, p<0.05			
					Serum MDA (μmol/L) (change from baseline): -3.43 (-6.02 to -0.83), p<0.05			
Hoseini et al. (2019) [55]	Renal function	Camel milk BID x 3 months	Routine care	MDRD	eGFR (pre vs post): 26.9±7.39 vs 31.45±8.99, p=0.001	Yes	16.9% (eGFR)	3 months
					SCr levels (pre vs post): 2.58±0.71 vs 2.2±0.48, p=0.01		-14.7% (SCr)	
					BUN (pre vs post): 60.31±22.61 vs 44.38±14.29, p=0.0001		-26.4% (BUN)	
Khajehdehi et al. (2011) [56]	Renal function	140 mg silymarin TID x 3 months	Placebo tablet TID x 3 months	ELISA	Proteinuria (mg/24h) (pre vs post, patients with type 2 diabetic nephropathy): 4328.7±3038.2 vs 2354.7±1800.1, p=0.001	Yes	-45.6% (proteinuria)	2 months
					IL-8 (pg/mL) (pre vs post, patients with type 2 diabetic nephropathy): 99.1±97.9 vs 43.6±55.0, p=0.002		-56.0% (IL-8)	
					TGF-β (pg/mL) (pre vs post, patients with overt type 2 diabetic nephropathy): 522.3±189.2 vs 397.3±55.2, p=0.006		-23.9% (TGB-β)	
					IL-8 (pg/mL) (pre vs post, patients with overt type 2 diabetic nephropathy): 41.4±50.3 vs 30.6±75.2, p=0.02		-26.1% (IL-8)	
Makhlough et al. (2010) [57]	Uremic pruritus	0.03% capsaicin ointment QID x 4 weeks	Placebo ointment QID x 4 weeks	Uremic pruritus scoring questionnaire by Duo	Pruritus score (T vs C): 2.5±2.5 vs 7.2±5.5, p<0.05	Yes	-84.3% (pruritus score)	
Pingali et al. (2020) [58]	Hyperuricemia	T_1_:500 mg of beleric capsule taken QD. T_2_: 1000 mg of beleric capsule taken QD	40 mg of Febuxostat taken QD	Jaffe method, MDRD Study equation, Salbutamol challenge test, Ellman’s method, Chrono-log light transmittance aggregometry, Spectrometry, Colorimetric detection with Griess reagents	SCr (pre vs post): group B: 1.86±0.32 vs 1.64±0.29, p≤0.005. Group C: 2.06±0.26 vs 1.56±0.24, p≤0.0001	Yes	-11.70%±9.00 (SCr, group B), -24.42%±8.14 (SCr, group C).	6 months
					eGFR (pre vs post): group B: 39.13±6.57 vs 45.96±11.14, p≤0.005. Group C: 34.78±5.34 vs 48.93±11.46, p≤0.0001		16.96%±14.87 (eGFR, group B), 40.39%±20.98 (eGFR, group C)	
					Serum uric acid (pre vs post): Group B:8.10±0.67 vs 6.46±0.34, p≤0.0001. Group C: 8.54±0.64 vs 5.63±0.37, p≤0.0001		19.84%±6.43 (serum uric acid, group B), 33.88%±4.95 (serum uric acid, group C)	
Naturopathy (n=5)								
Khan et al. (2014) [60]	Malnutrition	Alpha-keto amino acid tablets TID x 3 months	Placebo tablets TID x 3 months	Blood tests	Hb% (g/dL) (T vs C): 9.39±0.87 vs 8.91±1.48, p<0.05	Yes	19.8% (Hb%)	3 months
					FBG (mg/dL) (T vs C): 104.00±8.46 vs 113.78±14.31, p<0.001		-20.8% (FBG)	
					Blood urea (mg/dL) (T vs C): 66.07±19.29 vs 79.78±24.79, p<0.001		-38.1% (blood urea)	
					SCr (mg/dL) (T vs C): 2.83±1.10 vs 3.33±1.37, p<0.05		-39.5% (SCr)	
					24 h TUP (g/day) (T vs C): 2.06±0.61 vs 2.43±0.97, p<0.01		-38.3% (TUP)	
					24 Hour TUV (mL/day) (T vs C): 1943.23±204.1 vs 1736.76±176.04, p<0.001		33.3% (TUV)	
					GFR (mL/min) (T vs C): 29.4±3.68 vs 23.3±1.63, p<0.001		49.2% (GFR)	
Prakash et al. (2004) [61]	Malnutrition	Keto amino acid tablets QD x 9 months	Placebo tablets QD x 9 months	99mTc-DTPA plasma sample method	GFR (mL/min/ 1.73 m^2^) (pre vs post within C): 28.6±17.6 vs 22.5±15.9, p=0.015.	Progress of renal failure prevented.	-	9 months
					Serum total proteins (g%) (pre vs post within C): 7.04±0.66 vs 6.56±0.83, p=0.038			
					Mid-arm circumference (cm) (pre vs post within C): 28.0±4.4 vs 27.3±4.8, p=0.048			
Sedaghattalab et al. (2021) [62]	Inflammation	Watercress extract QD x 1 month	Placebo extract QD x 1 month	Blood tests, TBA reaction assay, Colorimetric kits, Spectrophotometer	BUN (mg/dL) (pre vs post): 40.6±11.2 vs 34.6±15.1, p<0.04.	Yes	-14.8% (BUN)	1 month
					Calcium (mg/dL) (pre vs post): 8.8±1.32 vs 10.4±2, p<0.001		18.1% (calcium)	
					Total oxidant status (μM) (pre vs post): 11.3±3.3 vs 6.9±2.4, p<0.001		-38.9% (total oxidant status)	
					Sulfhydryl protein (mmol/L) (pre vs post): 13.1±5.3 vs 7.4±4.3, p<0.001		-43.5% (sulfhydryl protein)	
					MDA (mmol/L) (pre vs post): 1.6±0.13 vs 0.42±0.27, p<0.001		-73.8% (MDA)	
					Superoxide dismutase (U/mL) (pre vs post): 29.3±6.3 vs 37.1±8.4, p<0.001		26.6% (superoxide dismutase)	
Zare et al. (2019) [63]	Inflammation	Garlic extract tablets TIW x 2 months	Placebo tablets TIW x 2 months	Human homocysteine kits, ELISA	IL-6 (pg/mL) (pre vs post): 2.2 (0.8, 6.4) vs 0.7 (0.6, 1.2), p<0.001	Yes	-68.2% (IL-6)	2 months
					CRP (mg/L) (pre vs post): 13.0 (5.0, 14.0) vs 2.0 (1.0, 9.0), p<0.001		-84.6% (CRP)	
					ESR (mm) (pre vs post): 50.7±28.5 vs 35.4±21.7, p=0.021.		-30.2% (ESR)	
Boldaji et al. (2019) [59]	Hypertension, stress, and inflammation	Pomegranate juice TIW x 2 months	Routine care	Mini nutritional assessment	MDA (μmol L^-1^) (pre vs post): 0.88±0.01vs 0.77±0.01, p<0.001	Yes	-12.5% (MDA)	2 months
					Total antioxidant capacity (mmol L^-1^) (pre vs post): 0.40±0.08vs 0.49±0.11, p<0.001		22.5% (total antioxidant capacity)	
					IL-6 (ng L^-1^) (pre vs post): 3.00±1.48 vs 2.09±1.25, p<0.0001		-30.3% (IL-6)	
Homeopathy (n=2)								
Cavalcanti et al. (2003) [16]	Uremic pruritus	Homeopathic verum medication^d^ administered	Placebo medication administered	Validated scale	Pruritus score (pre vs 15 vs 30 vs 45 vs 60 days): 65±25 vs 46±29, p=0.002 vs 41±30, p=0.002 vs 42±29, p=0.002 vs 38±33, p=0.004	Yes	-29.2% (pruritus score, pre vs 15 days), -36.9% (pruritus score, pre vs 30 days), -35.4% (pruritus score, pre vs 45 days), -41.5% (pruritus score, pre vs 60 days)	60 days
Silveira et al. (2019) [64]	Renal function	Brazilian green propolis pills BID x 3 months	Placebo pills BID x 3 months	Immunoturbidimetry, ELISA	Proteinuria (mg/24 h) (T vs C, baseline vs 12 months): 695 (95% CI, 483 to 999) vs. 1403 (95% CI, 1031 to 1909); p=0.004	Yes	-27.6% (proteinuria)	12 months

Tools used for outcomes assessments of symptoms during interventions are as follows: the most frequently utilized tools were the abbreviated version of the World Health Organization Quality of Life-100 Questionnaire (n=2) and visual analog scale (n=2).

Risk of bias within studies: Out of all included studies, 14 (42.4%) studies were assessed to be of "low" risk of bias, nine (27.3%) and 10 (30.3%) studies were scored as "some concerns" and "high" risk of bias, respectively.

Results of Individual Studies

Traditional Chinese medicine: The most commonly utilized interventions in the studies were herbal treatments (n=14, 70%) [17, 37-43, 45-49, 51, 52] followed by acupuncture (n=6, 30%) [3,7,36,38,44,50]. For acupuncture treatment, five studies used conventional acupuncture [3,7,36,38,50] while one study used infrared stimulation of acupoints [44]. The common acupoints administered during conventional acupuncture treatment were Li11 (n=3, 60%), ST36 (n=2, 20%), and Li4 (n=2, 20%). The frequency of acupuncture ranged from once a week to once a day, whereas the duration of studies lasted between six weeks to six months. Uremic pruritus (n=3, 60%) was the most commonly studied indication, with reductions in pruritus score observed between 54.7% and 60.2% [7,36,38]. Infrared stimulation was used on RN6, RN4, and RN3 thrice a week for three months [44]. The indication studied was quality of life (QoL). According to QoL scores that were evaluated using the EQ-5D questionnaire, utility increased by 15.2%.

Herbal treatments include six single-herb (n=6, 42.9%) [39-42,48,49,51] and eight multi-herbs formula granules (n=8, 57.1%) [17,37,41,43,45-47,52]. Common single-herb treatments used are Huangkui (n=2, 33.3%) and TWHF (n=2, 33.3%). The frequency of treatment was once to three times a day, for two to 12 months. Proteinuria was the most studied indication (n=4, 66.7%), where the various treatments reduced proteinuria between 27.0% and 61.8% [39,42,48,51]. Common herbs used in the multi-herb formula granules studies included Huang Qi (n=6, 75%), Danggui (n=4, 50%), *Salvia miltiorrhiza* (n=3, 37.5%), and Poria (n=3, 37.5%). The most commonly studied indication was improvement in renal function (n=5, 62.5%) [17,41,43,47,52]. Serum creatinine decreased (2.76-12.4%) and eGFR increased (7.07-15.5%) across the various treatments.

Ayurveda: The included studies evaluated both plant-based (n=3, 50%) [53,54,56] and animal-based (n=3, 50%) treatments [55,57,58]. The most common treatment studied was plant-based silymarin tablets (n=2, 33.3%) while that for clinical indication was improvement in renal function (n=4, 66.6%) [53-56]. The eGFR increased between 16.9% and 86.8% across the various treatments [53-56].

Naturopathy: Keto amino acids were the most studied naturopathic treatment (n=2, 40%). Common indications studied included anemia and glucose control, where hemoglobin and fasting blood glucose levels in subjects improved by 19.8% and 20.8%, respectively [60,61]. The use of watercress and garlic extract was also studied for inflammation, where the total oxidant status and IL-6 levels were shown to improve by 38.9% and 68.2%, respectively.

Homeopathy: Homeopathic verum was studied for relief of uremic pruritus with improvement in pruritus score between 29.2% and 41.5% [16]. For Brazilian green propolis pills, it was studied for proteinuria where a 27.6% reduction in proteinuria was noted [64].

Safety Profile of AMS Interventions

The adverse events reported by all included studies are shown in Table 3. Adverse events reported were of grade 1 (n=13, 39.4%), grade 2 (n=8, 24.4%), and grade 3 (n=6, 18.2%) severity. There were no life-threatening consequences or death related to adverse effects (grades 4 and 5) reported.

**Table 3:** Adverse effects reported with AMS usage. ^a^The adverse effects were graded based on Common Terminology Criteria for Adverse Effects (CTCAE). ^b^QDDHG is composed of Huang Qi, Danshen, Dihuang, Shanyao, and Gan Cao. ^c^Dosage and frequency vary from patient to patient. Grade 1: mild; asymptomatic or mild symptoms; clinical or diagnostic observations only; intervention not indicated. Grade 2: moderate; minimal, local, or non-invasive intervention indicated; limiting age-appropriate instrumental activities of daily living. Grade 3: severe or medically significant but not immediately life-threatening; hospitalization or prolongation of hospitalization indicated; disabling; limiting self-care activities of daily living. Grade 4: life-threatening consequences; urgent intervention indicated. Grade 5: death related to adverse effects; AE: adverse events; BID: twice a day; C: control arm; NA: not applicable; QD: once a day; T: treatment arm; TID: thrice a day; TIW: thrice a week

Study (year)	N (T)	Treatment	Reported adverse effects based on Common Terminology Criteria for Adverse Effects (CTCAE) v5.0^a,b,c^			Onset of adverse effects (if available)	Management and outcomes of patients
			Grade 1 (%)	Grade 2 (%)	Grade 3 (%)		
Traditional Chinese medicine (n=19)							
Chenet al. (2013) [37]	95	9.6 g of Shenqi particle TID x 48 weeks	NI	Interstitial pneumonia (n=1, 1.1%)	Lung infection (n=5, 5.26%); liver injury (n=3, 3.15%)	NI	NI
Geet al. (2013) [39]	34	40 mg Tripterygium Wilfordii Hook F TID x 3 months	Vomiting (n=13, 38.2%)	Hyperkalaemia (n=8, 23.5%); Leukopenia (n=1, 2.9%); Photosensitive dermatitis (n=3, 8.8%)	NA	NI	Patient with decreased white blood cell withdrawn from the study
Liet al. (2020) [42]	735	Huangkui capsule TID x 12 months	NA	NA	Upper respiratory tract infections (n=21, 2.9%)	NI	NI
Zhanget al. (2014) [51]	T_1_:133 T_2_:136	T_1_: Huangkui capsule TID x 6 months T_2_: Huangkui capsule TID and Losartan potassium tablet QD x 6 months	Elevated cholesterol (T1: n=5, 3.76%; T2: n=4, 2.94%)	Upper respiratory tract infections (T1: n=4, 3.0%; T2: n=4, 2.94%)	Liver injury (T1: n=3, 2.26%)	NI	NI
Xionget al. (2020) [48]	62	60 mg Tripterygium Wilfordii Hook F and 160 mg valsartan QD x 24 weeks	Itchy skin (n=4, 6.45%); nausea (n=3.22%); rash (n=1, 1.61%)	NA	Liver dysfunction (n=12, 19.4%); leukopenia (n=1, 1.61%)	NI	NI
Liet al. (2015) [41]	122	8 g Tangshen Formula granules and angiotensin receptor blockers BID x 24 weeks	NA	Anaemia (n=2)	Acute myocardial infarction (n=5)	NI	NI
Maet al. (2013) [43]	25	150 mL zishentongluo solution BID x 3 months	NA	NA	NA	NA	NA
Wanget al. (2012) [17]	T_1_:192 T_2_:191	T1: TCM granules BID x 24 weeks T_2_: TCM granules BID and 10 mg benazepril QD x 24 weeks	Dry cough (T_2_: n=2, 1.04%); Gastrointestinal symptoms (T_1_: n=7, 3.64%; T_2_: n=3, 1.57%)	Anaemia (T_1_: n=7, 3.64%; T_2_: n=6, 3.14%)	Liver injury (T_1_: n=2, 1.04%; T_2_: n=5, 2.61%); hyperkalaemia (T_1_ n=7, 3.64%; T_2_ n=18, 9.42%)	NI	NI
Yuet al. (2017) [50]	28	Acupuncture at Li4, ST36 and K13 acupoint QD x 3 months	Mild pain, bleeding and bruising in some patients	NA	NA	NI	Symptoms resolved spontaneously without any treatment
Zhaoet al. (2020) [52]	171	Herbal granules TID x 6 months	Mild abnormal liver function test (n=5, 2.92%); Mild discomfort (n=2, 1.17%)	NA		NI	NI
Xianget al. (2016) [47]	51	QDDHG^b^ tablets BID x 12 weeks	Insomnia (n=1, 1.96%)	NA	NA	NI	NI
Xuet al. (2016) [49]	91	500 mg GS-Rb1 (ginseng extract) QD x 6 months	NA	NA	NA	NI	NI
Che-yiet al. (2005) [36]	20	Acupuncture at Quchi (L11) acupoint TIW x 1 month	Elbow soreness (n=2; 10.0%)	NA	NA	NI	Symptoms resolved spontaneously after 1 day.
Gaoet al. (2002) [38]	34	Acupuncture at Quchi (L11) and Zusanli (ST 36) acupoint BIW x 1 month	NA	NA	NA	NA	NA
Nahidiet al. (2018) [7]	15	Acupuncture at various acupoints TIW x 6 weeks	NA	NA	NA	NA	NA
Onoet al. (2015) [3]	23	Acupuncture QIW x 2 months	NA	NA	NA	NA	NA
Suet al. (2009) [44]	31	Infrared stimulation of Qihai (RN6), Kuamyuan (RN4) and Chungchi (RN3) TIW x 3 months	NA	NA	NA	NA	NA
Wanget al. (2019) [45]	41	Qingshen granules TID x 3 months	NA	NA	NA	NA	NA
Wanget al. (2020) [46]	136	Qingshen granules TID x 3 months	NA	NA	NA	NA	NA
Liet al. (2009) [40]	32	Tianbaoning tablets (gingko extract) TID x 8 weeks	NA	NA	NA	NA	NA
Ayurveda (n=6)							
Alamet al. (2020) [53]	70	Sativa oil QD and alpha-keto amino acid tablets TID x 3 months	NA	NA	NA	NA	NA
Fallahzadehet al. (2012) [54]	30	140 mg silymarin tablet QD x 3 months	Nausea and vomiting (n=3, 10%); headache (n=2,6.67%)	NA	NA	NI	NI
Hoseiniet al. (2019) [55]	22	Camel milk BID x 3 months	NA	Abdominal pain (n=1, 4.5%)	NA	NI	NI
Khajehdehiet al. (2011) [56]	28	140 mg silymarin TID x 3 months	NA	NA	NA	NA	NA
Makhlough et al. (2010) [57]	17	0.03% capsaicin ointment QID x 4 weeks	NA	Severe skin burning (n=1, 2.8%)	NA	NI	NI
Pingaliet al. (2020) [58]	18	1000 mg of beleric capsule taken QD	Mild gastrointestinal intolerance (n=2, 11.1%)	NA	NA	NA	NA
Naturopathy (n=5)							
Khanet al. (2014) [60]	80	Alpha-keto amino acid tablets TID x 3 months	Nausea (n=5, 6.25%); diarrhoea (n=5, 6.25%)	NA	NA	NA	NA
Prakashet al. (2004) [61]	21	Keto amino acid tablets QD x 9 months	NA	NA	NA	NI	NI
Sedaghattalabet al. (2021) [62]	22	Watercress extract QD x 1 month	NA	NA	NA	NA	NA
Zareet al. (2019) [63]	19	Garlic extract tablets TIW x 2 months	NA	NA	NA	NI	NI
Boldajiet al. (2019) [59]	22	Pomegranate juice TIW x 2 months	Stomach discomfort (n=1, 4.54%)	NA	NA	NI	NI
Homeopathy (n=2)							
Cavalcantiet al. (2003) [16]	11	Homeopathic verum medication^c ^administered	NA	NA	NA	NA	NA
Silveiraet al. (2019) [64]	18	Brazilian green propolis pills BID x 3 months	NA	NA	NA	NA	NA

Adverse effects associated with traditional Chinese medicine: the use of acupuncture is associated with mild pain, bleeding, bruising, and elbow soreness (grade 1) [36,40]. These symptoms resolved spontaneously without additional treatment. For single-herb treatments, TWHF treatment was associated with vomiting (38.2%, grade 1), itchy skin (6.45%, grade 1), nausea (3.22%, grade 1), and rash (1.61%, grade 1) [39,48]. Grade 2 adverse events associated with its use included hyperkalemia (23.5%), leukopenia (2.9%), and photosensitive dermatitis (8.8%), while grade 3 adverse effects included liver dysfunction (19.4%) and severe leukopenia (1.61%). Subjects who developed leukopenia were withdrawn from the study.

For Huangkui treatment, adverse effects observed included elevated cholesterol (2.94-3.76%) (grade 1), upper respiratory tract infection (2.94-3%) (grade 2 and grade 3), and liver injury (2.26%) (grade 3) [42,51]. The use of Shenqi particles was associated with interstitial pneumonia (1.1%, grade 2), lung infection (5.26%, grade 3), and liver injury (3.15%, grade 3) [37]. Anemia (grade 2) and acute myocardial infarction (grade 3) were observed for subjects using Tangshen formula granules [41]. The use of QDDHG tablets was associated with insomnia (1.96%, grade 1) [47].

Adverse effects associated with ayurveda: Silymarin treatment was associated with nausea and vomiting (10%), as well as headache (6.67%) [54,56]. Beleric capsule treatment was associated with mild gastrointestinal intolerance (11.1%) [58]. Adverse effects from both studies were of grade 1 severity. Abdominal pain (4.5%, grade 2) was observed with the treatment of camel milk [55].

Adverse effects associated with naturopathy: All adverse effects reported for naturopathy treatments are of grade 1 severity (Table 3). They include nausea (6.25%) and diarrhea (6.25%) with the use of alpha-keto amino acid [60], as well as stomach discomfort (4.54%) with the use of pomegranate juice [59].

Adverse effects associated with homeopathy: No adverse effects were reported with the use of homeopathy (Table 3).

Summary of Efficacy and Safety Profile of AMS Interventions

Table 4 shows a summary related to the efficacy and safety profiles of AMS interventions.

**Table 4:** Summary of efficacy and safety profile of AMS indicated for CKD symptoms. ^a^QDDHG is composed of Huang Qi, Danshen, Dihuang, Shanyao, and Gan Cao. ^b^Dosage and frequency vary from patient to patient. Percentage reduction in symptoms is computed using (mean score at baseline - mean score post-treatment at end of study)/(mean score at baseline) for the treatment group. 99mTc-DTPA: 99 m technetium diethylenetri-aminepenta-aceticacid, BAID: brachial arterial inner diameter, BID: twice a day, ELISA: enzyme-linked immunosorbent assay, GS-Rb1: Ginseng extract, MDRD: Modification of Diet in Renal Disease, QoL: Quality of life, TBN: Tianbaoning, TID: thrice a day, TIW: thrice a week, TwHF: Tripterygium Wilfordii Hook F, ZSTL: zishentongluo

Type of AMS	Common doses and treatment regimens and duration of therapy	Indications	Percentage reduction in CKD symptoms (if available)	Adverse effects reported (%)
TCM (n=20) [3,7,17,36-52]	Herbal	Renal function	2.76% to 51.2%	Elbow soreness (10%); liver injury and dysfunction (1.04-19.40%); dry cough, pneumonia, and upper respiratory infections (1.04-5.26%); hyperkalemia (3.64-23.50%); vomiting (38.2%); leukopenia and anemia (1.61-2.90%); photosensitive dermatitis, itchy skin, and rash (1.65-8.8%); gastrointestinal symptoms, nausea, and vomiting (1.57-38.2%); insomnia (1.96%)
	Herbal granules TID x 6 months; QDDHG tablets^a^ BID x 12 weeks; 500 mg GS-Rb1 QD x 6 months; 8 g TSF granules and ARB BID x 24 weeks; 150 mL ZSTL solution BID x 3 months; TCM granules BID x 24 weeks			
	Acupuncture			
	Acupuncture at Li4, ST36 and K13 acupoint QD x 3 months			
	Herbal	Proteinuria	-61.8% to -18.6%	
	9.6 g of Shenqi particle TID x 48 weeks; 40 mg TWHF TID x 3 months followed by 20 mg TWHF TID x 3 months; Huangkui capsule TID x 12 months; 60 mg TWHF and 160 mg valsartan QD x 24 weeks			
	Acupuncture	Uremic pruritus	-60.2% to -54.7%	
	Acupuncture at Quchi (L11) acupoint TIW x 1 month; acupuncture at Quchi (L11) and Zusanli (ST 36) acupoint BIW x 1 month; acupuncture at various acupoints TIW x 6 weeks			
	Acupuncture QIW x 2 months	Fatigue, insomnia, itchiness, and pain	-89.2% to 15.2%	
	Infrared stimulation of Qihai (RN6), Kuamyuan (RN4) and Chungchi (RN3) TIW x 3 months	QoL	-9.10% to 163%	
	Qingshen granules TID x 3 months	Wnt/β-catenin signaling pathway	-61.4% to 17.8%	
	Qingshen granules TID x 3 months	Immune function	42.3% to -20.2%	
	TBN tablets (gingko extract) TI x 8 weeks	Vascular endothelial function	44.5% to 40.8%	
Ayurveda (n=6) [53-58]	Plant-based	Renal function	-56.0% to 86.8%	Nausea, vomiting, and headache (6.67-10%); abdominal pain (4.5%) severe skin burning (2.8%)
	Sativa oil QD and alpha-keto amino acid tablets TID; 140 mg silymarin TID x 3 months; 140 mg silymarin tablet QD x 3 months			
	Animal-based			
	Camel milk BID x 3 months			
	0.03% capsaicin ointment QID x 4 weeks	Uremic pruritus	-84.3%	
	1000/500 mg of beleric capsule taken QD	Hyperuricemia	-24.4% to 40.4%	
Naturopathy (n=5) [59-63]	Alpha-keto amino acid tablets TID x 3 months; Keto amino acid tablets QD x 9 months	Malnutrition	39.5% to 49.2%	Nausea (6.25%); diarrhea (6.25%); stomach discomfort (4.45%)
	Watercress extract QD x 1 month; garlic extract tablets TIW x 2 months	Inflammation	-73.8% to 26.6%	
	Pomegranate juice TIW x 2 months	Hypertension, stress, and inflammation	-30.3% to 22.5%	
Homeopathy (n=2) [16,64]	Homeopathic verum medication^b^ administered x 60 days; Brazilian green propolis pills BD x 3 months	Uremic pruritus	-41.5% to -29.2%	-
		Renal function	-27.6%	

Discussion

To the best of our knowledge, this is the first review that has summarized findings related to the therapeutic uses of AMS for CKD patients in RCTs. Among the four classes of AMS, TCM was the most studied class which has demonstrated efficacy in improving CKD-related symptoms and outcomes [3,7,36-52]. Among the TCM interventions evaluated, Huangkui, TWHF, and acupuncture have shown efficacy in reducing proteinuria and relieving uremic pruritus symptoms. The therapeutic basis of TCM for CKD is rooted in the restoration of vital energy and nourishment of blood, dispelling of heat and reduction of dampness, and regulation of Yin and Yang in the body [65]. In Western medicine, this is seen in a reduction in inflammation and oxidative stress, as well as boosting micro-circulation and enhancement of metabolism [52]. For example, Huangkui, also known as *Abelmoschus manihot*, reduces proteinuria by removing oxygen radicals, improving the circulation, and clearance of immune complexes as well as reducing inflammation and renal tubular epithelial injury [66]. It is also noted that triptolide, the key constituent of TWHF, suppresses the nuclear factor kappa b (NF-κB) signaling pathway and prevents the trigger of T lymphocytes and some inflammatory cytokines (TNF-α, IL-1β, IL-6, and IFN-γ), in addition to its podocyte-protective capabilities [67-70]. Notably, two of the included studies demonstrated that a combination of TCM and Western medicine, such as the intake of Huangkui capsule and losartan tablet to alleviate proteinuria, is more efficacious than taking TCM or Western medicine alone. This adds to existing evidence on potential applications of TCM alongside conventional medical therapy. Among patients on TWHF, regular checks of potassium and liver enzymes should be performed due to the risk of hyperkalemia and raised liver enzymes.

With regards to the use of acupuncture, it results in the release of endogenous opiate-like substances that have been proposed to dull the peripheral and central perception of itching [71]. Stimulation of acupoints via far infrared (FIR) treatments has also been revealed to boost skin microcirculation, lessen emotional anxiety and promote excretion of waste products by improving the autonomic nervous system [44,72-76]. Enhanced circulation via a stronger autonomic nervous system is postulated to relieve CKD-related symptoms as the development of renal failure is attributed to poor circulation in the field of TCM [73,77]. Currently, renowned hospitals in the United States such as the Mayo Clinic and Duke University Medical Center have started providing acupuncture, along with other treatments. With growing evidence related to the efficacy and safety of TCM, there has been greater receptivity from medical doctors related to applications and use of TCM in clinical practice [78]. It is however important to note that TCM is not without any side effects. For example, the use of Huangkui should be cautioned in patients with hyperlipidemia or liver disease as its use has been associated with elevated lipid levels and liver injury. On the other hand, acupuncture appears to be relatively safe with mild side effects, such as elbow soreness. More studies related to TCM are required to further assess their long-term safety profile, and they should be prescribed with careful consideration of each patient’s health condition.

For Ayurveda, silymarin was one of the most studied interventions which demonstrated efficacy in improving renal function. Ayurvedic therapies are derived predominantly from plants, animals, minerals, exercise, and lifestyle changes. They are believed to rejuvenate and remove toxins from one’s body. In conventional medicine, the therapeutic effects of ayurveda for CKD are ascribed to their anti-inflammatory and anti-oxidant properties. For instance, silymarin has shown efficacy in in vitro studies in attenuating inflammatory stress in renal tissue by suppressing the NF-κB signaling pathway and hence TNF production [79-85]. Other Ayurvedic treatments, such as the application of capsaicin ointment, were also found to alleviate uremic pruritus. Topical capsaicin, a natural alkaloid derived from red chili pepper, has been discovered to relieve uremic pruritus by binding specifically to type C sensory neurons and resulting in the release of substance P, as well as suppressing its synthesis, transport, and storage thereafter [57]. Relatively few side effects were observed for the Ayurvedic therapies discussed above. Despite the promising benefits associated with Ayurvedic treatments, it is currently less globally recognized as compared to TCM [86]. Further research is necessary to evaluate their efficacy and safety profile to improve their acceptance in clinical practice as adjunctive treatments, in particular for CKD patients.

For naturopathy, its unique attribute lies in the reprioritization of the order of therapeutics, with increased emphasis on non-invasive treatments, such as lifestyle modifications and nutrition, over medical or surgical interventions. In this review, one of the more studied interventions is the use of ketoanalogues of essential amino acids (KAs). The addition of KAs to a low-protein diet has been shown to improve renal function and uremia. Notably, while lowering protein intake may improve renal function in CKD patients by altering immunologic events and reducing hypertrophy and hyperfiltration in the remaining nephrons, it may result in malnutrition [87-89]. However, the supplementation of KAs not only averts malnutrition by ensuring adequate consumption of amino acids but also alleviates uremia [61]. The absence of amino nitrogen in KAs allows them to become transaminated by taking nitrogen from non-essential amino acids and hence, reducing the production of urea via re-using the amino group [90,91]. Relatively few and mild adverse effects were observed for the included naturopathic therapies, rendering them attractive treatment options. Additionally, as naturopathic treatments are usually non-invasive, they can be easily combined with conventional medications. Of note, 28 health systems, hospitals, and cancer treatment centers in the United States currently have at least one licensed naturopathic physician at their premises [92]. With increasing research evaluating the efficacy and safety profile of naturopathic treatments, its role as potential adjunctive treatment for CKD patients is also likely to expand in the future.

Lastly, homeopathy has also shown efficacy in improving CKD-related symptoms and outcomes. Homeopathy entails the therapeutic administration of substances derived from plants, minerals, or animals which produce effects that correspond to the clinical manifestation of diseases. In this review, the use of Brazilian green propolis pills and homeopathic verum medication were found to improve renal function and alleviate uremic pruritus, respectively. Brazilian green propolis was reported to improve renal function via a few mechanisms. Firstly, it decreases proteinuria via its ability to reduce urinary oxidative stress and macrophage infiltration into the kidneys [93]. Secondly, chrysin, a flavonoid in propolis has been shown to decrease podocyte apoptosis in patients with diabetic nephropathy and lessen glomerular injury [94]. Lastly, propolis has also been shown to decrease blood pressure via acetylcholine-induced vasodilation and from its antioxidant properties [93,95-97]. With regard to the safety of homeopathic treatment, no adverse effects were reported across included studies. However, the practice of homeopathy is relatively restricted, with 36% of states in the United States requiring homeopathic practitioners to either be licensed Western medicine or Naturopathic practitioners [32]. Consequently, more research is necessitated to validate the efficacy and safety of homeopathic treatment as adjunctive therapy for CKD patients.

Limitations

The following limitations should be considered in conjunction with this review. Firstly, due to the clinical and methodological heterogeneity of the studies, meta-analyses were not performed. As the pool of evidence for AMS trials for CKD patients grows, subsequent reviews should consider conducting meta-analyses for the efficacy of AMS treatments for CKD patients. In addition, there could have been exclusion of potentially applicable studies even though an extensive search strategy was used. To prevent this, the references of included studies were also hand-searched as part of our search strategy. Another limitation of the study relates to the inclusion of only articles in English language. Researchers should consider the inclusion of studies in other languages such as Chinese and Tamil in future reviews. Finally, although results of the included studies were reported normalized Z scores, care should be taken when interpreting these values and comparing the efficacy of various AMS classes. This is due to considerable diversity in types of outcomes evaluated and comparator arms and tools adopted for evaluation of outcomes. Overall, it is hoped that with greater standardization of study outcomes for AMS therapies in future studies, these normalized Z scores can enable more purposeful comparisons of the efficacy of the different AMS classes.

## Conclusions

In this review, TCM and naturopathy were the most studied AMS which have shown efficacy for indications, such as improvement in renal function, proteinuria, and uremic pruritus in CKD patients. Most studies recruited small number of patients and larger RCTs are necessary to continue assessing and validating these potential AMS therapies. Medical professionals who plan to incorporate the use of AMS in daily practice should tailor treatment based on each patient’s health condition and be cognizant of their associated adverse effects, if any.

## Appendices

Appendix 1: details of search strategy used

**Table 5:** Search strategy for MEDLINE.

S/N	Key terms	Search terms employed
1	Randomized controlled trials	({"Randomized controlled trial" (Publication Type) OR "randomized controlled trials as topic" (MeSH Terms) OR "randomized controlled trial" (All Fields) OR "randomized controlled trial" (All Fields)}) OR ({"random allocation" (MeSH Terms) OR ("random" {All Fields} AND "allocation" {All Fields}) OR "random allocation" (All Fields) OR "randomized" {All Fields}) AND controlled (All Fields) AND study (All Fields)} OR {"randomized controlled trial" (Publication Type) OR "randomized controlled trials as topic" (MeSH Terms) OR "randomized controlled trial" (All Fields) OR "randomized controlled trial" (All Fields)} OR {randomized (All Fields) AND controlled (All Fields) AND study (All Fields)})
2	Chronic kidney disease	({"Renal insufficiency, chronic"(MeSH Terms) OR ("renal"{All Fields} AND "insufficiency"{All Fields} AND "chronic"{All Fields}) OR "chronic renal insufficiency"(All Fields) OR ("chronic"{All Fields} AND "kidney"{All Fields} AND "disease"{All Fields} OR "chronic kidney disease"{All Fields}) OR ("renal insufficiency, chronic"{MeSH Terms} OR ("renal"{All Fields} AND "insufficiency"{All Fields} AND "chronic"{All Fields}) OR ("chronic renal insufficiency"{All Fields} OR ("chronic"{All Fields} AND "kidney"{All Fields} AND "insufficiency"{All Fields}) OR "chronic kidney insufficiency"{All Fields}) OR ("renal insufficiency, chronic"{MeSH Terms} OR "renal"{All Fields} AND "insufficiency"{All Fields} AND "chronic"{All Fields}) OR "chronic renal insufficiency"(All Fields) OR ("chronic"{All Fields} AND "renal"{All Fields} AND "disease"{All Fields}) OR "chronic renal disease"{All Fields} OR "kidney failure, chronic"(MeSH Terms) OR ("kidney"{All Fields} AND "failure"{All Fields} AND "chronic"{All Fields}) OR "chronic kidney failure"{All Fields} OR ("chronic"{All Fields} AND "renal"{All Fields} AND "disease"{All Fields}) OR ("renal insufficiency, chronic"{MeSH Terms} OR "renal"{All Fields} AND "insufficiency"{All Fields} AND "chronic"{All Fields}) OR "chronic renal insufficiency"{All Fields} OR ("chronic"{All Fields} AND "renal"{All Fields} AND "insufficiency"{All Fields}) OR ("renal insufficiency"(MeSH Terms) OR ("renal"{All Fields} AND "insufficiency"{All Fields}) OR "renal insufficiency"{All Fields} OR ("kidney"{All Fields} AND "failure"{All Fields}) OR "kidney failure"{All Fields}) OR ("renal insufficiency"(MeSH Terms) OR ("renal"{All Fields} AND "insufficiency"{All Fields}) OR "renal insufficiency"{All Fields} OR ("renal"{All Fields} AND "failure"{All Fields}) OR "renal failure"{All Fields}) OR ("kidney failure, chronic"(MeSH Terms) OR ("kidney"{All Fields} AND "failure"{All Fields} AND "chronic"{All Fields}) OR "chronic kidney failure"{All Fields} OR ("end"{All Fields} AND "stage"{All Fields} AND "renal"{All Fields} AND "failure"{All Fields}) OR "end stage renal failure"{All Fields}) OR ("kidney failure, chronic"(MeSH Terms) OR ("kidney"{All Fields} AND "failure"{All Fields} AND "chronic"{All Fields}) OR "chronic kidney failure"{All Fields} OR ("end"{All Fields} AND "stage"{All Fields} AND "renal"{All Fields} AND "disease"{All Fields}) OR "end stage renal disease"{All Fields}) OR predialysis{All Fields} OR pre-dialysis{All Fields} OR non-dialysis{All Fields} OR "renal dialysis"(MeSH Terms) OR ("renal"{All Fields} AND "dialysis"{All Fields}) OR "renal dialysis"{All Fields}) OR ("kidney diseases"{MeSH Terms} OR "kidney"{All Fields} AND "diseases"{All Fields}) OR "kidney diseases"{All Fields} OR ("kidney"{All Fields} AND "disease"{All Fields}) OR "kidney disease"{All Fields}) OR ("haemodialysis"{All Fields} OR "renal dialysis"{MeSH Terms}) OR ("renal"{All Fields} AND "dialysis"{All Fields}) OR "renal dialysis"{All Fields} OR "hemodialysis"{All Fields}) OR ("haemodialysis"{All Fields} OR "renal dialysis"{MeSH Terms}) OR ("renal"{All Fields} AND "dialysis"{All Fields}) OR "renal dialysis"({All Fields} OR "hemodialysis"{All Fields}) OR ("peritoneal dialysis"{MeSH Terms} OR ("peritoneal"{All Fields} AND "dialysis"{All Fields}) OR "peritoneal dialysis"{All Fields})
3	Manipulative and body-based methods	({Randomized controlled trial} OR {randomized controlled study} OR {randomized controlled trial} OR {randomized controlled study}) AND ({chronic kidney disease} OR {chronic kidney insufficiency} OR {chronic renal disease} OR {chronic renal insufficiency} OR {kidney failure} OR {renal failure} OR {end-stage renal failure} OR {end-stage renal disease} OR {predialysis} OR {pre-dialysis} OR {non-dialysis} OR {renal dialysis} OR {Kidney disease} OR {hemodialysis} OR {hemodialysis} OR {peritoneal dialysis}) AND ({Traditional Chinese Medicine} OR {Traditional Medicine} OR {Chinese Herbal Drugs} OR {Alternative Medicine} OR {Complementary Medicine} OR {Complementary therap*} OR {Alternative therap*} OR {Natural Medicine} OR {Integrative Medicine} OR TCM OR Ayurved* OR ayuverda OR Naturopath* OR naturopathy OR Homeopath* OR homeopathy OR {medicinal plant} OR {plant extract} OR {Plant Preparation} OR {Chinese medicinal herb} OR herb OR {natural remedies} OR {oriental medicine} OR {Materia Medica} OR {Diet-based therap*} OR {ethnopharmacology OR ethnomedicine OR kampo OR kanpo OR herbology})

**Table 6:** Search strategy for Embase.

S/N	Key terms	Search terms employed
1	Randomized controlled trials	(“Randomized controlled trial”/exp OR “randomized controlled trial” OR {randomized AND controlled AND (“trial”/exp OR trial)} OR “randomized controlled study”/exp OR “randomized controlled study” OR {randomized AND controlled AND (“study”/exp OR study)} OR “randomized controlled trial”/exp OR “randomized controlled trial” OR {randomized AND controlled AND (“trial”/exp OR trial)} OR “randomized controlled study”/exp OR “randomized controlled study” OR {randomized AND controlled AND (“study”/exp OR study)})
2	Chronic kidney disease	(“Chronic kidney disease”/exp OR “chronic kidney disease” OR {chronic AND (“kidney”/exp OR kidney) AND (“disease”/exp OR disease)} OR “chronic kidney insufficiency”/exp OR “chronic kidney insufficiency” OR {chronic AND (“kidney”/exp OR kidney) AND insufficiency} OR “chronic renal disease”/exp OR “chronic renal disease” OR {chronic AND (“renal”/exp OR renal) AND (“disease”/exp OR disease)} OR “chronic renal insufficiency”/exp OR “chronic renal insufficiency” OR {chronic AND (“renal”/exp OR renal) AND insufficiency} OR “kidney failure”/exp OR “kidney failure” OR {(“kidney”/exp OR kidney) AND (“failure”/exp OR failure)} OR “renal failure”/exp OR “renal failure” OR {(“renal”/exp OR renal) AND (“failure”/exp OR failure)} OR “end-stage renal failure”/exp OR “end-stage renal failure” OR {“end stage” AND (“renal”/exp OR renal) AND (“failure”/exp OR failure)} OR “end-stage renal disease”/exp OR “end-stage renal disease” OR {“end stage” AND (“renal”/exp OR renal) AND (“disease”/exp OR disease)} OR “predialysis”/exp OR predialysis OR “pre dialysis” OR “non dialysis” OR “renal dialysis”/exp OR “renal dialysis” OR {(“renal”/exp OR renal) AND (“dialysis”/exp OR dialysis)} OR “kidney disease”/exp OR “kidney disease” OR {(“kidney”/exp OR kidney) AND (“disease”/exp OR disease)} OR “hemodialysis”/exp OR hemodialysis OR “hemodialysis”/exp OR hemodialysis OR “peritoneal dialysis”/exp OR “peritoneal dialysis” OR {peritoneal AND (“dialysis”/exp OR dialysis)})
3	Manipulative and body-based methods	(“Alternative medicine”/exp OR “alternative medicine” OR {alternative AND (“medicine”/exp OR medicine)} OR “alternative therapy”/exp OR “alternative therapy” OR {alternative AND (“therapy”/exp OR therapy)} OR “complementary medicine”/exp OR “complementary medicine” OR {complementary AND (“medicine”/exp OR medicine)} OR “complementary therapy” OR {complementary AND (“therapy”/exp OR therapy)} OR “natural medicine” OR {(“natural”/exp OR natural) AND (“medicine”/exp OR medicine)} OR “integrative medicine”/exp OR “integrative medicine” OR {integrative AND (“medicine”/exp OR medicine)} OR “massage”/exp OR massage OR chiropractice OR “yoga”/exp OR yoga OR “tai chi”/exp OR “tai chi” OR {tai AND chi} OR “moxibustion”/exp OR moxibustion OR “cupping”/exp OR cupping OR “qi gong”/exp OR “qi gong” OR {(“qi”/exp OR qi) AND gong} OR “acupuncture”/exp OR acupuncture OR osteopath* OR naprapath* OR reflexo* OR “shiatsu”/exp OR shiatsu OR “tui na”/exp OR “tui na” OR {tui AND (“na”/exp OR na)}) AND (“spinal manipulation”/exp OR “spinal manipulation” OR {spinal AND (“manipulation”/exp OR manipulation)})

**Table 7:** Search strategy for CINAHL. CINAHL: Cumulative Index to Nursing and Allied Health Literature

S/N	Key terms	Search terms employed
1	Randomized controlled trials	({Randomized controlled trial} OR {randomized controlled study} OR {randomized controlled trial} OR {randomized controlled study})
2	Chronic kidney disease	({Chronic kidney disease} OR {chronic kidney insufficiency} OR {chronic renal disease} OR {chronic renal insufficiency} OR {kidney failure} OR {renal failure} OR {end-stage renal failure} OR {end-stage renal disease} OR {predialysis} OR {pre-dialysis} OR {non-dialysis} OR {renal dialysis} OR {Kidney disease} OR {hemodialysis} OR {hemodialysis} OR {peritoneal dialysis})
3	Manipulative and body-based methods	({Alternative medicine} OR {Alternative therapy} OR {Complementary Medicine} OR {Complementary therapy} OR {Natural Medicine} OR {Integrative Medicine} OR {Massage} OR {Chiropractice} OR yoga OR {Tai Chi} OR moxibustion OR cupping OR {Qi Gong} OR Acupuncture OR Osteopath* OR Naprapath* OR Reflexo* OR Shiatsu OR {Tui Na} {Spinal manipulation})

**Table 8:** Search strategy for PsycINFO.

S/N	Key terms	Search terms employed
1	Randomized controlled trials	({Randomized controlled trial} OR {randomized controlled study} OR {randomized controlled trial} OR {randomized controlled study})
2	Chronic kidney disease	({Chronic kidney disease} OR {chronic kidney insufficiency} OR {chronic renal disease} OR {chronic renal insufficiency} OR {kidney failure} OR {renal failure} OR {end-stage renal failure} OR {end-stage renal disease} OR {predialysis} OR {pre-dialysis} OR {non-dialysis} OR {renal dialysis} OR {Kidney disease} OR {hemodialysis} OR {hemodialysis} OR {peritoneal dialysis})
3	Manipulative and body-based methods	({Alternative medicine} OR {Alternative therapy} OR {Complementary Medicine} OR {Complementary therapy} OR {Natural Medicine} OR {Integrative Medicine} OR {Massage} OR {Chiropractice} OR yoga OR {Tai Chi} OR moxibustion OR cupping OR {Qi Gong} OR Acupuncture OR Osteopath* OR Naprapath* OR Reflexo* OR Shiatsu OR {Tui Na} {Spinal manipulation})

**Table 9:** Search strategy for Scopus.

S/N	Key terms	Search terms employed
1	Randomized controlled trials	(TITLE-ABS-KEY {({randomized AND controlled AND trial} OR {randomized AND controlled AND study} OR {randomized AND controlled AND trial} OR {randomized AND controlled AND study})})
2	Chronic kidney disease	(TITLE-ABS-KEY {({randomized AND controlled AND trial} OR {randomized AND controlled AND study} OR {randomized AND controlled AND trial} OR {randomized AND controlled AND study })}) AND (TITLE-ABS-KEY {({chronic AND kidney AND disease } OR {chronic AND kidney AND insufficiency} OR {chronic AND renal AND disease} OR {chronic AND renal AND insufficiency} OR {kidney AND failure} OR {renal AND failure} OR {end-stage AND renal AND failure} OR {end-stage AND renal AND disease} OR {predialysis} OR {pre-dialysis} OR {non-dialysis} OR {renal AND dialysis} OR {kidney AND disease} OR {hemodialysis} OR {hemodialysis} OR {peritoneal AND dialysis})})
3	Manipulative and body-based methods	(TITLE-ABS-KEY {(alternative AND medicine) OR (complementary AND medicine) OR (complementary AND therapy) OR (alternative AND therapy) OR (natural AND medicine) OR (integrative AND medicine) OR (massage) OR (chiropractice) OR yoga OR (tai AND chi) OR (moxibustion) OR (cupping) OR (qi AND gong) OR (acupuncture) OR (Osteopath*) OR (Naprapath*) OR (Reflexo*) OR (Shiatsu) OR (Tui Na) OR (Spinal manipulation)})

**Table 10:** Search strategy for Cochrane Central Register of Controlled Trial (CENTRAL).

S/N	Key terms	Search terms employed
1	Randomized controlled trials	({Randomized controlled trial} OR {randomized controlled study} OR {randomized controlled trial} OR {randomized controlled study})
2	Chronic kidney disease	({Chronic kidney disease} OR {chronic kidney insufficiency} OR {chronic renal disease} OR {chronic renal insufficiency} OR {kidney failure} OR {renal failure} OR {end-stage renal failure} OR {end-stage renal disease} OR {predialysis} OR {pre-dialysis} OR {non-dialysis} OR {renal dialysis} OR {Kidney disease} OR {hemodialysis} OR {hemodialysis} OR {peritoneal dialysis})
3	Manipulative and body-based methods	({Alternative medicine} OR {Alternative therapy} OR {Complementary Medicine} OR {Complementary therapy} OR {Natural Medicine} OR {Integrative Medicine} OR {Massage} OR {Chiropractice} OR yoga OR {Tai Chi} OR moxibustion OR cupping OR {Qi Gong} OR Acupuncture OR Osteopath* OR Naprapath* OR Reflexo* OR Shiatsu OR {Tui Na} {Spinal manipulation})

Modeled after: https://journals.plos.org/plosone/article?id=10.1371/journal.pone.0223564

Appendix 2: risk of bias assessment

**Table 11:** Risk of bias assessment for AMS studies.

Study (year of publication)	Domain 1: risk of bias from the randomization process	Domain 2: risk of bias due to deviations from the intended interventions	Domain 3: missing outcome data	Domain 4: risk of bias in measurement of the outcome	Domain 5: risk of bias in selection of the reported result	Overall Risk of bias judgment
Traditional Chinese medicine (n=20)						
Li et al. (2015) [41]	Low	Low	Low	Low	Low	Low
Ma et al. (2013) [43]	Some concerns	Low	Low	Low	Low	Low
Wang et al. (2012) [17]	Low	Low	High	Low	Low	Some concerns
Yu et al. (2017) [50]	Low	High	Low	Low	Low	High
Zhao et al. (2020) [52]	Low	Low	Some concerns	Low	Low	Some concerns
Xiang et al. (2016) [47]	Low	Some concerns	Low	Low	Low	Low
Xu et al. (2016) [49]	Low	Low	Low	Low	Low	Low
Chen et al. (2013) [37]	Low	Low	Low	Low	Low	Low
Ge et al. (2013) [39]	Some concerns	Some concerns	Low	Low	Low	Low
Li et al. (2020) [42]	Some concerns	Low	Some concerns	High	Low	Some concerns
Zhang et al. (2014) [51]	Some concerns	Low	Low	Low	Low	Some concerns
Xiong et al. (2020) [48]	Low	Some concerns	Low	Low	Low	Low
Che-Yi et al. (2005) [36]	Low	Low	Low	Low	Low	Low
Gao et al. (2002) [38]	Some concerns	Some concerns	Low	Low	Low	Low
Nahidi et al. (2018) [7]	Low	High	High	Low	Low	High
Ono et al. (2015) [3]	Low	High	High	High	Low	High
Su et al. (2009) [44]	Some concerns	Some concerns	Some concerns	Some concerns	Low	High
Wang et al. (2019) [45]	Low	Low	Low	Low	Low	Low
Wang et al. (2020) [46]	Low	High	High	Low	Low	High
Li et al. (2009) [40]	Low	Some concerns	Low	Low	Low	Low
Ayurveda (n=6)						
Alam et al. (2020) [53]	Low	Some concerns	Low	Low	Some concerns	Some concerns
Fallahzadeh et al. (2012) [54]	Low	Low	Low	Low	Some concerns	Some concerns
Hoseini et al. (2019) [55]	Low	Some concerns	Some concerns	Low	Some concerns	Some concerns
Khajehdehi et al. (2011) [56]	Low	Low	Low	Low	Low	Low
Makhlough et al. (2010) [57]	Low	Low	Low	Low	Low	Low
Pingali et al. (2020) [58]	Low	Low	Low	Low	Low	Low
Naturopathy (n=5)						
Khan et al. (2014) [60]	Low	Low	Low	Low	Low	Low
Prakash et al. (2004) [61]	Low	Low	Low	Low	Low	Low
Sedaghattalab et al. (2021) [62]	Low	Low	Low	Low	Low	Low
Zare et al. (2019) [63]	Low	Low	Low	Low	Low	Low
Boldaji et al. (2019) [59]	Low	Low	Low	Low	Low	Low
Homeopathy (n=2)						
Cavalcanti et al. (2003) [16]	Low	Low	High	Low	Low	High
Silveira et al. (2019) [64]	Low	Low	Low	Low	Low	Low
